# Fluorescence sensing of metal ions in solution using a morpholine-containing phenolic Mannich base of 1′-hydroxy-2′-acetonaphthone†

**DOI:** 10.1039/d4ra07200f

**Published:** 2024-12-06

**Authors:** Andreea Laura Chibac-Scutaru, Gheorghe Roman

**Affiliations:** a Petru Poni Institute of Macromolecular Chemistry, Department of Polyaddition and Photochemistry Iaşi 700487 Romania andreea.chibac@icmpp.ro; b Petru Poni Institute of Macromolecular Chemistry, Department of Inorganic Polymers Iaşi 700487 Romania gheorghe.roman@icmpp.ro

## Abstract

A phenolic Mannich base derived from 1′-hydroxy-2′-acetonaphthone (HAN) as a substrate and morpholine as an amine reagent was synthesized and structurally characterized. The sensing ability toward various metal ions of the s-, p- and d-block of this molecule that has the binding site for metal ions in the starting *ortho*-hydroxyphenone preserved was examined. Interaction between this phenolic Mannich base and Al^3+^, Cr^3+^, Cu^2+^ and Co^2+^ leads to modifications of the sensing molecule's absorption spectrum. Fluorescence spectroscopy showed that Al^3+^ acts as a fluorescence enhancer, whereas Cu^2+^ functions as a fluorescence quencher for the aminomethylated derivative. The phenolic Mannich base may be employed either as a sensitive “turn-on” chemosensor for Al^3+^ or as a sensitive “turn-off” chemosensor for Cu^2+^. However, in the presence of these ions at identical concentrations, the Mannich base becomes a selective chemosensor for Al^3+^. The sensing ability of this phenolic Mannich base toward rare earth ions showed that Eu^3+^, Dy^3+^ and Gd^3+^ induce changes in the absorption spectrum of the Mannich base. Fluorescence spectroscopy showed that the response of the sensing molecule toward Eu^3+^ and Dy^3+^ is weak, and this phenolic Mannich base may be used as a “turn-off” chemosensor for these two lanthanide ions only in a narrow concentration range (1–16 × 10^−5^ M).

## Abstract 2

A phenolic Mannich base derived from 1′-hydroxy-2′-acetonaphthone 1 was synthesised. It demonstrated the ability to act as a fluorescent sensor for Al^3+^, exhibiting a “turn-on” response, and for Cu^2+^, exhibiting a “turn-off” response.

## Graphical abstract

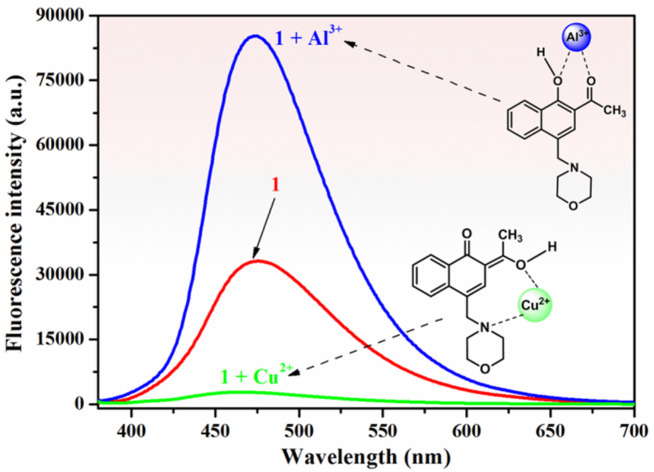

## 1 Introduction

A plethora of applications for the investigative optical spectroscopic technique based on the fluorescent properties of a sensing molecule have become available and have been extensively used in the last two decades for qualitative and quantitative measurements of minute amounts of various distinct analytes, such as metal ions (especially heavy metal ions), anions, biotic molecules, or toxic inorganic or organic compounds in both environmental and biological samples.^1–9^ As part of fluorescence spectroscopy, these applications are rapid, simple, sensitive, very often highly selective, non-invasive and cost-efficient, but they require suitable sensing molecules that can be either discovered through screening of potential fluorophore-containing chemical entities or specifically designed and subsequently developed for a particular analyte. While the use of some of these fluorophore-containing probes in fluorescent detection is well-established (for example, in the case of pyrazolines,^10,11^ 4-bora-3a,4a-diaza-*s*-indacene (BODIPY),^12,13^ crown ethers^14^ and other macrocyles,^15^ or fluorescein^16^), other molecules whose structure would potentially allow them to act as fluorescent sensors are still overlooked. For example, *ortho*-hydroxyaryl ketones have been known to form luminescent complexes with lanthanides^17,18^ and boron^19,20^ using spatially adjacent hydroxyl and carbonyl groups for the generation of coordination compounds. A large number of derivatives of these *ortho*-hydroxyphenones (particularly those in whose structure the oxygen of the carbonyl group is replaced with nitrogen) have been disclosed as fluorescent chemosensors.^21–30^ Nevertheless, the chemosensor applications of *ortho*-hydroxyphenones and their derivatives whose structures preserve the binding site for metal ions remain basically underexplored, considering the small number of reports dealing with this topic.^31–34^ Nonetheless, 1-(1-hydroxynaphthalen-2-yl)ethanone (also known as 1′-hydroxy-2′-acetonaphthone or HAN) has been recently disclosed as a highly selective and sensitive “turn-on” sensor for Al^3+^ in pure water, while a series of other cations had no influence on the fluorescence intensity of this probe.^35^ It should be noted that this particular probe works only at pH values higher than 5. In addition to its newly reported ability to detect Al^3+^, 1-(1-hydroxynaphthalen-2-yl)ethanone has been studied both experimentally and computationally to ascertain the existence of an excited-state intramolecular proton transfer (ESIPT) process.^36–41^ To the best of our knowledge, aminomethylation of phenolic substrates has been scarcely used to produce chemosensors for metal ions so far. Moreover, all these probes present an aminomethyl group *ortho* to the phenolic hydroxyl in their structure and rely on the proximity of the hydroxyl and amino functions for the coordination of the metal ions.^42–44^ However, aminomethyl groups attached to various fluorophores represent the hallmark for fluorescent photoinduced electron transfer (PET) “off–on” signaling systems,^45^ which are employed mostly as switching receptors for pH;^46,47^ examples exist in which this type of probe detects cations,^48^ trace of water^49^ or saccharides.^50^ Among others, aminomethylation has been used to render hydrophilic substrates soluble in water by taking advantage of the capability of the amino function in the resulting Mannich bases to form salts with acids. Amines with various structures have been employed in the generation of Mannich bases.^51^ Besides being one of the most common amine reagents in aminomethylations, which has been shown to provide facile access in good to excellent yields to the desired reaction products,^52^ morpholine is also a structural motif easily recognized in the structure of fluorescent probes with rhodamine,^53–56^ quinoline,^57,58^ naphthalimide,^59^ tetraphenylethylene,^60^ triphenylamine,^61^ coumarin,^62^ fluorescein,^63^ BODIPY,^64^ or phthalocyanine^65^ scaffolds. These scaffolds have been substituted with morpholine because of its involvement as an efficient electron donor moiety in intramolecular PET processes,^65^ or as an electron acceptor moiety to quench the fluorescence of the fluorophores *via* d-PET.^64^

To broaden the knowledge concerning the applications of *ortho*-hydroxyphenones in the detection of metal ions, and considering the recently reported application of 1-(1-hydroxynaphthalen-2-yl)ethanone in sensing Al^3+^, this study aims to investigate the influence that the substitution of the aforementioned molecule through aminomethylation using morpholine as an amine reagent has on the efficiency of detecting metal ions. The synthesis through a facile procedure of a phenolic Mannich base derived from 1-(1-hydroxynaphthalen-2-yl)ethanone and its subsequent structural characterization, along with a thorough investigation of the sensing ability of the resulting 1-(1-hydroxy-4-(morpholinomethyl)naphthalen-2-yl)ethanone toward a significant number of diverse metal ions to determine this chemosensor's selectivity and sensitivity and to garner information in support of a tentative mechanism of detection are reported herein.

## 2 Experimental section

### 2.1. Materials and instrumentation

**Fig. 1:**
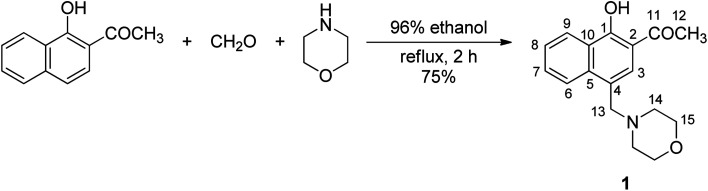
Synthetic approach to phenolic Mannich base 1.

1′-Hydroxy-2′-acetonaphthone, morpholine, formaldehyde solution (37 wt% in water), Pb(CH_3_COO)_2_·3H_2_O, Cu(CH_3_COO)_2_·H_2_O, Co(CH_3_COO)_2_·4H_2_O, Cd(CH_3_COO)_2_·2H_2_O, Hg(CH_3_COO)_2_·3H_2_O, CH_3_COONa·2H_2_O, CH_3_COOAg, MnCl_2_·4H_2_O, AlCl_3_, Cr(NO_3_)_3_·9H_2_O, Zn(NO_3_)_2_·6H_2_O, Ni(NO_3_)_2_·6H_2_O, Ca(NO_3_)_2_·4H_2_O, FeSO_4_·7H_2_O, Fe(NO_3_)_3_·9H_2_O, Ce(NO_3_)_3_·6H_2_O, La(NO_3_)_3_·6H_2_O, Gd(NO_3_)_3_·6H_2_O, Eu(NO_3_)_3_·5H_2_O, Dy(NO_3_)_3_·5H_2_O, Sm(CH_3_COO)_3_·4H_2_O, and ethylenediaminetetraacetic acid disodium salt dehydrate (EDTA) were purchased from Merck-Sigma-Aldrich (Germany). Methanol was provided by Honeywell Riedel-de Haën (Germany), while 96% ethanol was a product of Chemical Company (Romania). All chemical reagents and solvents were used without prior purification. The melting point was determined using a Mel Temp II apparatus and was uncorrected. NMR spectra were recorded using a Bruker Avance NEO spectrometer operating at 400 MHz, with a 5 mm probe for direct detection of ^1^H, ^13^C, ^19^F and ^29^Si. The spectra were recorded at room temperature using the standard parameter sets provided by Bruker. The residual signal of chloroform in CDCl_3_ was used as an internal standard (*δ* = 7.26 ppm for ^1^H, and *δ* = 77.0 ppm for ^13^C). The assignment of the signals in the NMR spectra of compound 1 was based on additional 2D homo- and hetero-nuclear correlation experiments (H,H-COSY, H,C-HSQC and H,C-HMBC). For NMR analysis purposes, the numbering of the structures reported in this study is illustrated in Fig. 1. Elemental analysis was performed on a Vario EL III CHNS analyzer. The electronic absorption spectrum (UV-vis) of compound 1 was recorded in methanol, at a concentration of 10^−4^ M, at room temperature, using a SPECORD 210 Plus Analytik Jena spectrophotometer. Fluorescence spectra (*C* = 10^−4^ M compound 1) were recorded using an RF-6000 Shimadzu spectrofluorometer in methanol at room temperature, using the excitation wavelength and the absorption maximum identified in the UV-vis spectrum of this compound (*λ*_ex_ = 365 nm). Aqueous solutions of the inorganic compounds employed in the detection experiments were prepared by dissolving the required amount of particular metal salt in distilled water with the aim of generating a solution containing 3 × 10^−3^ M cations. The absolute values of the fluorescence quantum yield (*Φ*) were measured using an FLS980 fluorospectrometer with an integrating sphere. The measurements were performed in dilute solutions (*A* < 0.1) using 10 mm quartz cuvettes, and a nanosecond diode laser centered at 365 nm was used as the excitation source. The fluorescence lifetimes were measured using a time-correlated single-photon counting spectrometer (FLS980, Edinburgh Instruments) with a nanosecond diode laser centred at 375 nm used as the excitation source. The fluorescence lifetimes were obtained by fitting the decay data to the multiexponential model. The best-fitted parameters were estimated by minimizing the reduced chi-square (*χ*^2^) value and the experimental data's residual distribution. Results having *χ*^2^ values around 1 and symmetrical distributions of the residual data were accepted.

### 2.2. Synthesis of 1-(1-hydroxy-4-(morpholinomethyl)naphthalen-2-yl)ethanone 1

A solution containing 1′-hydroxy-2′-acetonaphthone (2.79 g, 15 mmol), morpholine (2.61 g, 30 mmol), and aq. formaldehyde solution (37% by weight, 3 mL, 34 mmol) in 96% ethanol (15 mL) was heated at reflux temperature for 2 h. The mixture was refrigerated overnight, and the solid that had separated was filtered, washed with a mixture of 2-propanol–hexanes (2 × 10 mL, 1 : 1, v/v), air-dried, and recrystallized from 96% ethanol to afford 3.21 g (75%) yellow crystals, mp 126–127 °C. ^1^H NMR (CDCl_3_, 400.13 MHz), *δ* (ppm): 2.50 (t, *J* = 4.2 Hz, 4H, H14), 2.71 (s, 3H, H12), 3.69 (t, *J* = 4.6 Hz, 4H, H15), 3.80 (s, 2H, H13), 7.53–7.57 (m, 2H, H3 and H8), 7.68 (t, *J* = 7.8 Hz, 1H, H7), 8.25 (d, *J* = 8.3 Hz, 1H, H6), 8.49 (d, *J* = 8.3 Hz, 1H, H9), 14.00 (s, 1H, OH). ^13^C NMR (CDCl_3_, 100.6 MHz), *δ* (ppm): 26.9 (C12), 53.6 (C14), 61.6 (C13), 67.1 (C15), 112.3 (C2), 123.8 (C4), 124.7 (C9), 124.9 (C6), 125.6 (C10), 125.8 (C3), 126.0 (C8), 130.0 (C7), 136.5 (C5), 162.4 (C1), 204.1 (C11). Anal. calcd for C_17_H_19_NO_3_, %: C, 71.56; H, 6.71; N, 4.91. Found: C, 71.82; H, 6.90; N, 4.66.

### 2.3. UV-vis and fluorescence spectroscopy experiments

For UV-vis spectroscopy experiments, the solution containing compound 1 (3 mL) was placed in a standard UV quartz spectrometer cuvette cell with a path length of 1 cm. UV-vis and detection experiments followed the change in the absorption pattern of the spectrum upon the addition of 500 μL of an aqueous solution of a particular metal ion (3 × 10^−3^ M).

The fluorescence detection experiments examined the modification of the fluorescence spectrum of compound 1 (3.0 mL methanol solution, *C* = 10^−4^ M) produced by the addition of an aqueous solution of a particular metal ion (200 μL, *C* = 3 × 10^−3^ M). The standard deviation of the blank measurement used to determine LOD (eqn (1)) was calculated by recording the fluorescence spectrum of compound 1 ten times. The UV-vis and fluorescence titration measurements using compound 1 were performed by stepwise addition of 1–700 μL of a 3 × 10^−3^ M aqueous solution of selected metal ions (Al^3+^, Cu^2+^, Co^2+^, Eu^3+^ and Dy^3+^).

## 3 Results and discussion

### 3.1. Synthesis, structural and optical characteristics of phenolic Mannich base 1

To conserve the binding site for metal ions in HAN, chemical modification of this substrate was performed in the aromatic hydrocarbon fragment of the molecule. Aminomethylation of this phenolic ketone with two potential active sites (at the carbon atom α relative to the carbonyl function and at the carbon atom *para* to the hydroxyl group) can be achieved chemoselectively through the judicious selection of the reaction conditions.^66^ Accordingly, the use of morpholine as free amine and of formaldehyde in the form of its aqueous solution as the two requisite reagents in aminomethylation allowed access to the phenolic Mannich base 1 (Fig. 1) with very good yields (84%) of a material deemed sufficiently pure by NMR analysis for most purposes, but which was recrystallized to secure the required purity for spectroscopic applications. The structure of compound 1 was confirmed through NMR spectroscopy (Fig. S1–S5 in ESI†). Its ^1^H NMR spectrum showed two triplets centered at 2.50 ppm and 3.69 ppm, each integrating four protons attributed to the magnetically equivalent hydrogen atoms in the methylene groups of morpholine adjacent to nitrogen and oxygen, respectively. The hydrogen atoms in the methylene group bridging morpholine and the naphthalene ring system appear as a singlet integrating two protons at 3.80 ppm. The presence of a singlet at 2.71 ppm integrating for three protons assigned to the hydrogen atoms of the methyl group, along with the existence of only six aromatic protons, proved that aminomethylation occurred in the naphthalene ring system, and not in the methyl group next to the carbonyl function. The phenolic hydrogen atom is associated with the singlet in the off set, and the high value of its chemical shift value (14.00 ppm) is a consequence of its involvement in an intramolecular hydrogen bonding with the oxygen atom from the neighboring carbonyl function. The success of the aminomethylation is further substantiated by identifying the three peaks (53.6 ppm, 61.6 ppm and 67.1 ppm) in the aliphatic region of the ^13^C NMR spectrum of compound 1 associated with the carbon atoms in the morpholinomethyl fragment.

**Fig. 2:**
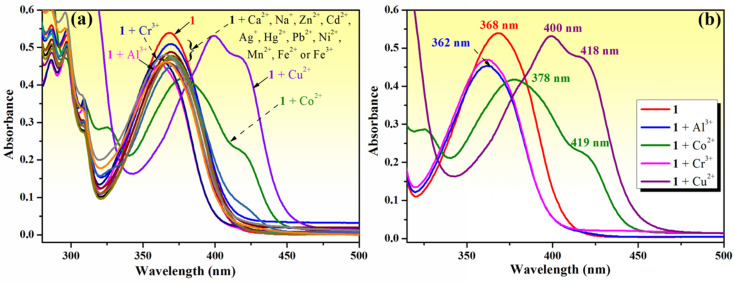
(a) Change in the absorption spectrum of compound 1 (10^−4^ M) in the presence of 5 equivalents of different metal ions (Na^+^, Ag^+^, Ca^2+^, Zn^2+^, Mn^2+^, Cu^2+^, Co^2+^, Ni^2+^, Cd^2+^, Hg^2+^, Pb^2+^, Fe^2+^, Fe^3+^, Al^3+^, and Cr^3+^) recorded in methanol–water (85 : 15, v/v); (b) details of shifts in the absorption maximum of phenolic Mannich base 1 (10^−4^ M) following the addition of 5 equivalents of Cu^2+^, Co^2+^, Al^3+^ or Cr^3+^ ions.

The UV-vis spectrum of compound 1 in methanol (10^−4^ M) exhibits a strong, broad absorption band at 368 nm (which is assigned to the state corresponding to an intramolecularly hydrogen-bonded closed enol conformer similar to that of the starting material HAN),^35,39^ and three sharp bands at 310, 298 and 287 nm (Fig. 2a). When compared to the HAN, the absorption bands of 1 exhibit a minor red shift and an increase in the molar extinction coefficient (Table S1 in ESI†). The fluorescence spectrum of phenolic Mannich base 1 (10^−4^ M in methanol) exhibits upon excitation with *λ*_ex_ = 365 nm a moderate emission at 475 nm, with slightly better quantum yield compared to HAN (Table S1 in ESI†).

### 3.2. Sensing of various metal cations by phenolic Mannich base 1

The chemosensing ability of compound 1 (10^−4^ M) for several metal cations (Na^+^, Ag^+^, Ca^2+^, Zn^2+^, Mn^2+^, Cu^2+^, Co^2+^, Ni^2+^, Cd^2+^, Hg^2+^, Pb^2+^, Fe^2+^, Fe^3+^, Al^3+^ and Cr^3+^) was initially assessed using UV-vis spectroscopy. The modification of the absorption spectrum of compound 1 upon the addition of 5 equivalents of tested metal ions is shown in Fig. 2a. The addition of Na^+^, Ag^+^, Ca^2+^, Zn^2+^, Mn^2+^, Ni^2+^, Cd^2+^, Hg^2+^, Pb^2+^, Fe^2+^ or Fe^3+^ ions did not cause a significant change in the absorption band profile, while the presence of Cu^2+^, Co^2+^, Al^3+^ or Cr^3+^ ions induced shifts and changed the profile of the absorption curve. Upon closer inspection, it can be observed that the addition of Al^3+^ or Cr^3+^ ions results in a hypsochromic shift of the absorption peak from 368 to 362 nm along with a decrease in absorption intensity (Fig. 2b), which is suggestive of the existence of an interaction between phenolic Mannich base 1 and Al^3+^ or Cr^3+^ ions, leading to the formation of coordinative compounds. In the case of the addition of Co^2+^ and Cu^2+^ ions, important red shifts of the initial absorption band of compound 1 to 378 nm (for Co^2+^) and to 400 nm (for Cu^2+^), along with the appearance of new absorption bands centered at 418–419 nm, were tentatively attributed to the binding affinity of compound 1 in the ground state for Co^2+^ and Cu^2+^ and to the existence of charge transfer in complexes 1–Cu^2+^ and 1–Co^2+^.

**Fig. 3:**
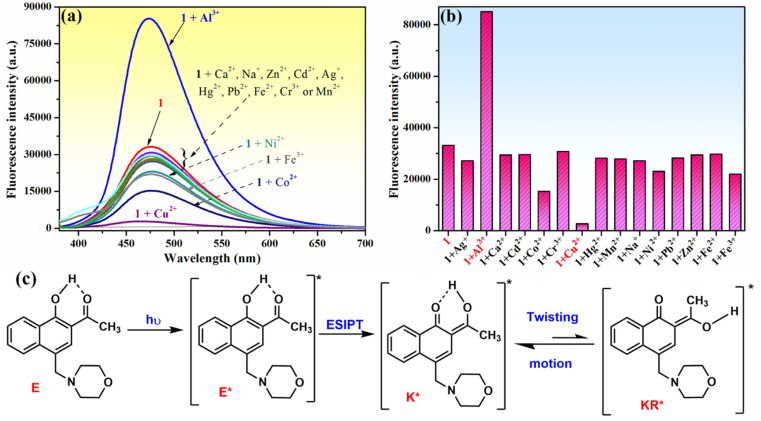
(a) Change in the fluorescence spectrum and (b) fluorescence intensity of compound 1 (10^−4^ M) in the presence of 2 equivalents of different metal ions (Na^+^, Ag^+^, Ca^2+^, Zn^2+^, Mn^2+^, Cu^2+^, Co^2+^, Ni^2+^, Cd^2+^, Hg^2+^, Pb^2+^, Fe^2+^, Fe^3+^, Al^3+^, and Cr^3+^) recorded in methanol–water (85 : 15, v/v) at *λ*_ex_ = 365 nm; (c) molecular structures of different prototautomers of phenolic Mannich base 1, and its ESIPT process triggered by photoexcitation.

Because the fluorescence technique is more sensitive than the UV-vis investigations, we next examined the ability of compound 1 to function as a fluorescence sensor to detect metal ions. The moderate fluorescence emission at 475 nm of phenolic Mannich base 1 (10^−4^ M in methanol) (Fig. 3a) has been assigned to the excited keto tautomers (K*) of compound 1 formed as a result of a fast excited state intramolecular proton transfer (ESIPT) process in the excited enol tautomer (E*) of the phenolic Mannich base (Fig. 3c).^39,67^ Malini *et al.* commented on a considerable reduction in the fluorescence emission of HAN owing to the possibility of a twisting motion between K* and KR* prototautomers in the excited state associated with its ESIPT process.^35^ Because the part of HAN's structure involved in the ESIPT process is highly conserved in the structure of compound 1, a behavior similar to that of HAN in terms of fluorescence may be expected from compound 1. However, when compared to HAN, phenolic Mannich base 1 shows a slight enhancement of fluorescence properties (Table S1 in ESI†), which we hypothesize is a consequence of the presence of the morpholinomethyl group attached to HAN's scaffold. The aminomethylation appears to have a marginal impact on the ESIPT process, presumably by slightly diminishing the twisting motion in compound 1. The fluorescence behavior of compound 1 indicates that it may be used for the development of a simple fluorescence chemosensor with dual “turn-on” and “turn-off” responses. Next, the chemosensing ability of compound 1 (10^−4^ M) toward several metal ions (2 equivalents of Na^+^, Ag^+^, Ca^2+^, Zn^2+^, Mn^2+^, Cu^2+^, Co^2+^, Ni^2+^, Cd^2+^, Hg^2+^, Pb^2+^, Fe^2+^, Fe^3+^, Al^3+^ and Cr^3+^) was evaluated using fluorescence spectroscopy, and the results obtained are presented in Fig. 3a and b. Thus, the presence of some of the aforementioned metal ions in the sample containing compound 1 excited at 365 nm only affects the fluorescence intensity of compound 1, but not the position of its emission maximum at *λ*_em_ = 475 nm (Fig. 3a). Of all the cations examined in this study, only Al^3+^, Cu^2+^ and Co^2+^ ions have a significant effect on the fluorescence intensity of compound 1. Upon the addition of aqueous Cu^2+^ solution (2 equivalents) to the solution of Mannich base 1, the latter's fluorescence emission is almost completely quenched, whereas the addition of Al^3+^ ions (2 equivalents) causes an enhancement in the emission intensity of compound 1 up to almost 3 times. The results show that phenolic Mannich base 1 is a highly sensitive fluorescence sensor with a dual response, namely “turn-off” for Cu^2+^ ions and “turn-on” for Al^3+^ ions. The amplification of fluorescence of sensing molecule 1 in the presence of Al^3+^ might be attributed to chelation that impedes the excited-state tautomeric interconversion process between K* and KR* tautomers.^35^ Additionally, this fluorescence enhancement observed for compound 1 in the presence of Al^3+^ ions may be due to the latter's electron configuration, which does not usually entail any electron- or energy-transfer mechanisms for the deactivation of an excited state.^68^ The possible reason for quenching the fluorescence of compound 1 by Cu^2+^ ions might be the paramagnetic character and also the incomplete d shell of Cu^2+^ ions, which determine an electron or energy transfer from 1 to Cu^2+^ upon complexation.^69–71^

**Fig. 4:**
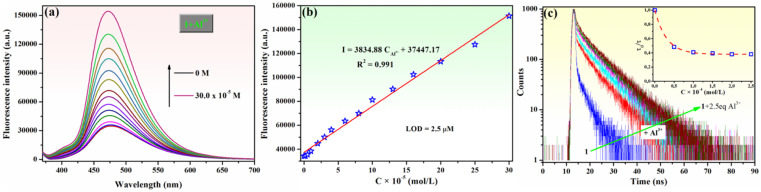
(a) Fluorescence spectra and (b) linear relationship plot of fluorescence intensity (*λ*_em_ = 475 nm) of compound 1 (10^−4^ M) upon addition of different concentrations of Al^3+^ ions (0–3 equivalents) when *λ*_ex_ = 365 nm. (c) Emission decays of compound 1 (10^−4^ M) in the absence and presence of various Al^3+^ ion concentrations (0.5–2.5 × 10^−4^ M). The inset shows the change in the lifetime of 1 at different concentrations of Al^3+^ ions. Samples were excited at 375 nm, and emission decays were measured at 475 nm.

The relationship between the fluorescence intensity of phenolic Mannich base 1 at 475 nm (*λ*_ex_ = 365 nm) and various concentrations of Al^3+^ ions was also studied (Fig. 4a and b). The fluorescence intensity of 1 was enhanced by increasing the concentration of the considered analyte (Fig. 4a), and a suitable linear relationship between the fluorescence emission intensity of compound 1 and Al^3+^ concentration (0–3 equivalents) was obtained, as illustrated in Fig. 4b. This indicates that chemosensor 1 is an adequate probe for the quantitative determination of Al^3+^ ions using the linear regression equation *I* = 3834.88*C*_Al_^3+^ + 37 447.17, with a correlation coefficient *R*^2^ = 0.991, where *C*_Al_^3+^ value determined from the equation is Al^3+^ concentration (×10^−5^ mol L^−1^).

The detection limit (LOD) of compound 1 for the Al^3+^ ions was calculated using eqn (1):

1 LOD = 3*σ*/*s*,

where *σ* is the standard deviation of blank measurement and *s* is the slope of the linear fitting curve of the sample fluorescence *versus* the metal ion concentration.^72^ The LOD value obtained to determine Al^3+^ ions by phenolic Mannich base 1 is 2.5 × 10^−6^ M, which is lower than the acceptable limit set by the World Health Organization (WHO) for Al^3+^ ions in drinking water (7.4 × 10^−6^ M),^73^ making fluorescent sensor 1 very sensitive toward Al^3+^ ions. The “turn-on” response of 1 could be interpreted as a result of an impediment of both the ESIPT process and twisting motion of molecule 1 upon chelation with Al^3+^, which suggests that the aforementioned response of sensor 1 occurs *via* chelation-enhanced fluorescence (CHEF).^74,75^ This is also confirmed by the fluorescence decay behavior of 1 (*C* = 10^−4^ M) obtained in the presence of different concentrations of Al^3+^ ions (Fig. 4c). The fluorescence decay of compound 1 fits well with a bi-exponential function with two-lifetime components. The major component (83.12%), with a lifetime value (*τ*_1_) of approximately 0.238 ns, was attributed to the prototautomeric keto form (K*) of 1,^35^ while the minor component (16.88%), having a lifetime value (*τ*_2_) of approximately 4.151 ns, was assigned to the twisted keto rotamer form (KR*) of 1,^35^ illustrating an effective average lifetime (*τ*_F_) of 1 to 3.289 ns (Table S2 in ESI†). As the concentration of Al^3+^ ions increases gradually, both the initial part of the decay process and the rate of decay in the tail part slow down. Ultimately, as can be observed from the inset of Fig. 4c, the overall decay rate decreases significantly and reaches a point of near saturation at a concentration of Al^3+^ ions of approximately 10^−4^ M, which is accompanied by a more than two-fold increase in the effective average lifetime (*τ*_F_ = 8.053 ns). The observed increase in both lifetime components (*τ*_1_ of K* keto form and *τ*_2_ of KR* tautomer of 1 bound to Al^3+^ ions, *τ*_1_ = 0.379 ns and *τ*_2_ = 8.208 ns) in comparison to the lifetime components of K* and KR* forms of free 1 (Table S2 in ESI†) indicates that Al^3+^ is bound to both species in the excited state. However, as can be observed in the same table, the percentage contribution (*A*_1_) of the *τ*_1_ component corresponding to the K* form decreases after the addition of Al^3+^ to compound 1, while the percentage contribution (*A*_2_) of the *τ*_2_ component assigned to the KR* prototautomer increases from 16.88% (for 1) to almost 70% (at *C*_Al_^3+^ = 10^−4^ M). This is interpreted as an indication that the equilibrium between the K* and KR* tautomers of compound 1 in the excited state shifts towards the latter for the 1–Al^3+^ system, as previously observed for a similar derivative.^35^

**Fig. 5:**
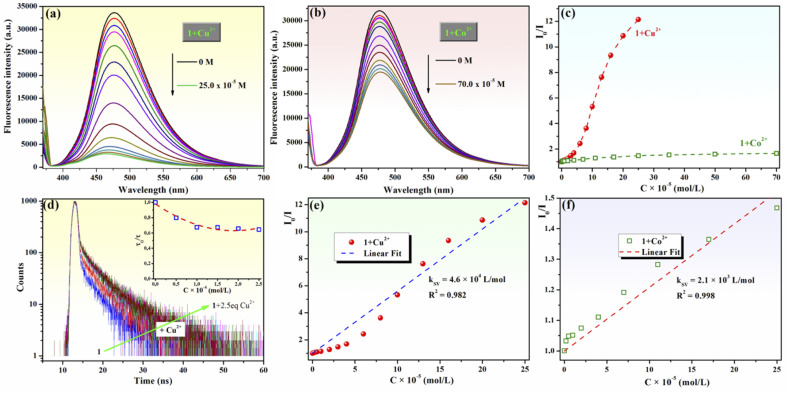
Fluorescence spectra of compound 1 (10^−4^ M) upon addition of different concentrations of (a) Cu^2+^ ions (0–2.5 equivalents) and (b) Co^2+^ ions (0–7 equivalents). (c) Stern–Volmer plots for the interactions between compound 1 and Cu^2+^ or Co^2+^ ions (*λ*_ex_ = 365 nm). (d) Emission decays of compound 1 (10^−4^ M) in the absence and presence of various Cu^2+^ ion concentrations (0.5–2.5 × 10^−4^ M). The inset shows the change in the lifetime of 1 at different concentrations of Cu^2+^ ions. Samples were excited at 375 nm, and emission decays were measured at 475 nm. Linear fit of Stern–Volmer plots for the interactions between compound 1 and Cu^2+^ (d) or Co^2+^ (e) ions at concentration intervals of 0–25 × 10^−5^ M (*λ*_ex_ = 365 nm).

Next, the fluorescence quenching behavior of chemosensor 1 upon the gradual addition of increasing amounts of aqueous Cu^2+^ (0–2.5 equivalents) or Co^2+^ (0–7 equivalents) solutions was investigated (Fig. 5). The fluorescence emission of Mannich base 1 was almost completely quenched when 2.5 equivalents Cu^2+^ (Fig. 5a) were added to the sample, whereas a fluorescence quenching of only 39.4% was observed upon the addition of 7 equivalents Co^2+^ (Fig. 5b). Based on these experiments, the detection limit of compound 1 calculated according to eqn (1) is 3.5 × 10^−6^ M for Cu^2+^ and is 16.6 × 10^−6^ M for Co^2+^. The detection limit of compound 1 for Cu^2+^ ions is below WHO's admissible concentration (30.0 × 10^−6^ M) for this ion in drinking water. As no information on the detection of Cu^2+^ ions by HAN has been disclosed previously,^35^ the ability of compound 1 to sense Cu^2+^ represents an obvious advantage over 1-(1-hydroxynaphthalen-2-yl)ethanone.

Fluorescence titrations of 1 with Cu^2+^ and Co^2+^ ions were employed to generate the Stern–Volmer plots (Fig. 5c), which were obtained based on eqn (2):

2 *I*_0_/*I* = 1 + *K*_SV_[Q],

where *I*_0_ is the initial fluorescence intensity of the solution of compound 1, *I* is the fluorescence intensity after the addition of the quencher, *K*_SV_ is the Stern–Volmer quenching constant, and [Q] is the concentration of the quencher. The sensitivity and selectivity of compound 1 for Cu^2+^ detection during the quenching process are demonstrated by the Stern–Volmer plots, as depicted in Fig. 5c, showing much greater efficiency in quenching the fluorescence of 1 by Cu^2+^ ions compared to Co^2+^ ions. The Stern–Volmer graph for Cu^2+^ presents an upward curvature, followed by a linear domain, suggesting that fluorescence quenching occurs through a combination of static and dynamic mechanisms. Furthermore, an examination of the fluorescence decay behavior of 1 (*C* = 10^−4^ M, *τ*_F_ = 3.289 ns) in the presence of different concentrations of Cu^2+^ ions (Fig. 5d) reveals that the overall decay rate slows down with increasing concentration of Cu^2+^ ions, reaching almost a plateau value of *τ*_F_ = 4.87 ns at a Cu^2+^ concentration of approximately 10^−4^ M (inset of Fig. 5d and Table S3 in ESI†). Additionally, a more pronounced increase in the second lifetime component (*τ*_2_) of the complex of KR* tautomer of 1 with Cu^2+^ ions was observed (5.364 ns at *C*_Cu_^2+^ = 10^−4^ M), which was accompanied by an increase in its percentage contribution (*A*_2_ = 33.55%), as shown in Table S3 (ESI).† However, the contribution of *τ*_1_, corresponding to the complex of K* of 1 with Cu^2+^ ions (*A*_2_ = 66.45%) remains significant, which seems to point to an equilibrium between the K* and KR* tautomers in the excited state for the 1–Cu^2+^ system. Hence, Cu^2+^ ions interact with 1 in an excited state, resulting in an energy transfer from the excited state of 1 to Cu^2+^ with non-radiative deactivation of the excited state, which in turn leads to a reduction in fluorescence intensity and a prolongation of the excited state lifetime. This behavior is consistent with dynamic quenching as the dominant mechanism for the interaction between compound 1 and Cu^2+^ ions.

The Stern–Volmer constants (*K*_SV_) for the fluorescence titration of compound 1 with Cu^2+^ and Co^2+^ were determined as the slopes of the linear fit of the plots (Fig. 5e and f), and their values are *K*_SV_ = 4.6 × 10^4^ L mol^−1^ for Cu^2+^ (*R*^2^ = 0.982) and *K*_SV_ = 2.1× 10^3^ L mol^−1^ for Co^2+^ (*R*^2^ = 0.998). The Stern–Volmer constant for Cu^2+^ ions is one order of magnitude higher than that for Co^2+^ as a quencher, demonstrating that compound 1 is selective and more sensitive for Cu^2+^ detection by fluorescence quenching than for any other cations evaluated in this report. This result could be the consequence of a chelation-enhanced quenching (CHEQ) effect of Cu^2+^ ions^75,76^ on the fluorescence of compound 1.

### 3.3. Selectivity and sensitivity of phenolic Mannich base 1 for Al^3+^ ions by “turn-on” response and for Cu^2+^ ions by “turn-off” response

**Fig. 6:**
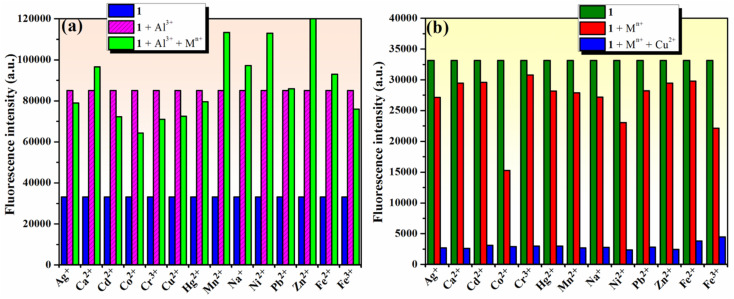
(a) Selectivity of compound 1 (10^−4^ M) towards Al^3+^ (2 equivalents) in competition experiments in the presence of 2 equivalents of other investigated metal ions (Na^+^, Ag^+^, Ca^2+^, Zn^2+^, Mn^2+^, Cu^2+^, Co^2+^, Ni^2+^, Cd^2+^, Hg^2+^, Pb^2+^, Fe^2+^, Fe^3+^, and Cr^3+^). (b) Selectivity of compound 1 (10^−4^ M) towards Cu^2+^ (2 equivalents) in the presence of 2 equivalents of other metal ions, except for Al^3+^ (Na^+^, Ag^+^, Ca^2+^, Zn^2+^, Mn^2+^, Co^2+^, Ni^2+^, Cd^2+^, Hg^2+^, Pb^2+^, Fe^2+^, Fe^3+^, and Cr^3+^); (*λ*_ex_ = 365 nm).

To estimate the selectivity of compound 1 for Al^3+^ ion in a “turn-on” response and for Cu^2+^ in a “turn-off” response over other metal ions, competition experiments using either Al^3+^ or Cu^2+^ ions and other metal ions were carried out using fluorescence spectroscopy. The results in Fig. 6a show that the fluorescence intensity of 1 (10^−4^ M) in the presence of 2 equivalents of Al^3+^ ions was only marginally affected in the presence of 2 equivalents of competing metal ions (Na^+^, Ag^+^, Ca^2+^, Zn^2+^, Mn^2+^, Cu^2+^, Co^2+^, Ni^2+^, Cd^2+^, Hg^2+^, Pb^2+^, Fe^2+^, Fe^3+^, and Cr^3+^). These results suggest that phenolic Mannich base 1 could be used as a selective “turn-on” fluorescent chemosensor for Al^3+^. The results for the competitive experiments involving 1 (10^−4^ M) and 2 equivalents of Cu^2+^ ions in the presence of other metal ions (2 equivalents of Na^+^, Ag^+^, Ca^2+^, Zn^2+^, Mn^2+^, Cu^2+^, Co^2+^, Ni^2+^, Cd^2+^, Hg^2+^, Pb^2+^, Fe^2+^, Fe^3+^, or Cr^3+^) in Fig. 6b demonstrate that 1 could be useful as a selective “turn-off” fluorescent chemosensor for Cu^2+^ in the presence of the other metal ions in excess, except for Al^3+^.

Job's method^77^ was employed to determine the stoichiometry between compound 1 and either Al^3+^ or Cu^2+^ ions in the respective complexes formed in the solution. The changes in fluorescence intensity were plotted as a function of the mole fraction of Al^3+^/Cu^2+^ ions (Fig. S6a and b in ESI†) and the binding stoichiometry results indicated that both Al^3+^ and Cu^2+^ ions form 1 : 1 complexes with the sensing compound 1. Presumably, the remaining positive charges of these metal ions in their complexes with compound 1 are counterbalanced by the corresponding anions (Cl^−^ in the case of Al^3+^ and CH_3_COO^−^ in the case of Cu^2+^) from the inorganic salts employed in the assay. In addition, one could not exclude the participation of solvent molecules (either methanol or water) through their oxygen atoms as electron donors to the metal ion in the structure of the complexes.

The association constant of phenolic Mannich base 1 for “turn-on” sensing of Al^3+^ could be calculated using the Benesi–Hildebrand equation.^75,78^ Meanwhile, for the fluorescence “turn-off” observed for Cu^2+^, the binding constant could be calculated by the slightly modified Benesi–Hildebrand equation.^75,79^ All details for these calculations are presented in the ESI† associated with this article. The association constants (*K*_a_) for complexes 1–Al^3+^ and 1–Cu^2+^, determined as the ratio of intercept and slope of the plots depicted in Fig. S7a and b (ESI),† are 1.91 × 10^3^ L mol^−1^ and 7.77 × 10^3^ L mol^−1^, respectively.

**Fig. 7:**
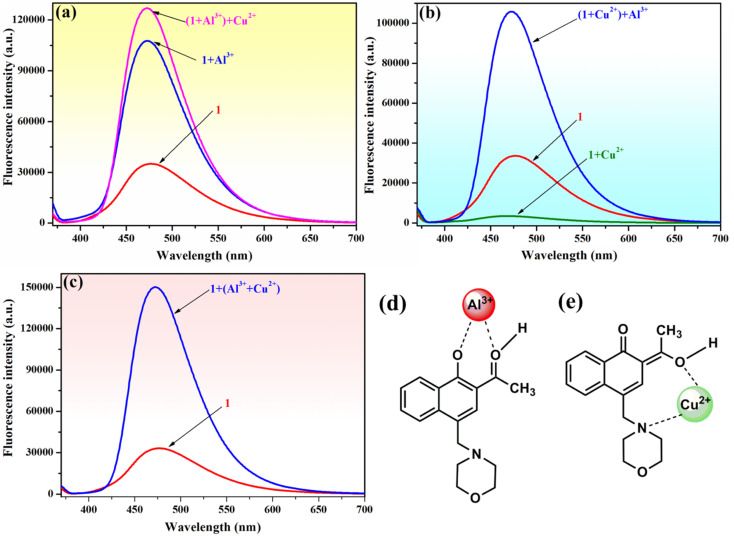
Behavior of the initial fluorescence intensity of compound 1 (10^−4^ M) upon mixing with Al^3+^ (2 equivalents) and Cu^2+^ (2 equivalents) ions in methanol–water (85 : 15, v/v) (*λ*_ex_ = 365 nm) when (a) Al^3+^ is added first, followed by Cu^2+^; (b) Cu^2+^ is added first, followed by Al^3+^; and (c) a mixture of (Al^3+^ + Cu^2+^) is added. Structures of complexes 1–Al^3+^ (d) and 1–Cu^2+^ tentatively proposed with a view to explaining the selectivity of compound 1 toward Al^3+^ in the presence of Cu^2+^.

To verify the selectivity and sensitivity of phenolic Mannich base 1 in a system containing both Al^3+^ and Cu^2+^ ions, the fluorescence intensity of compound 1 (10^−4^ M) was measured after adding 2 equivalents of Al^3+^ and Cu^2+^ ions sequentially to the system. As presented in Fig. 7, three distinct experiments were designed and performed as follows: (i) Al^3+^ was added first, followed by Cu^2+^ (Fig. 7a); (ii) Cu^2+^ was added first, followed by Al^3+^ (Fig. 7b); and (iii) a mixed solution of (Al^3+^ + Cu^2+^) was added to the sample of sensor 1 (Fig. 7c). Interestingly, the fluorescence intensity of 1 is enhanced in all these experiments, even in the presence of Cu^2+^ ions. The selectivity of compound 1 toward Al^3+^ in the presence of Cu^2+^ could tentatively be attributed to the different complexation modes of these two metal ions with the ligand. Compound 1 and Al^3+^ most likely form a ground-state complex with the structure depicted in Fig. 7d, which is similar to the structure previously supported by Malini *et al.* in the case of HAN.^35^ We hypothesize that the morpholine unit plays an important role in the interaction of compound 1 with Cu^2+^ ions, which results in the latter's detection through a “turn-off” response. We propose that compound 1 and Cu^2+^ form a complex in the excited stated, and the assumed structure of the complex may involve an interaction between Cu^2+^ and the nitrogen atom of morpholine (Fig. 7e). In addition, it is important to note that the formation of the 1–Al^3+^ complex is more facile sterically than the formation of the 1–Cu^2+^ complex, which may also contribute to the selectivity of compound 1 toward Al^3+^ in the presence of Cu^2+^. Thus, phenolic Mannich base 1 could be used as a highly selective and sensitive “turn-on” fluorescence sensor for the Al^3+^ ion over other metal species.

### 3.4. Response time and reversibility of sensor 1 for the detection of Al^3+^ and Cu^2+^ ions

**Fig. 8:**
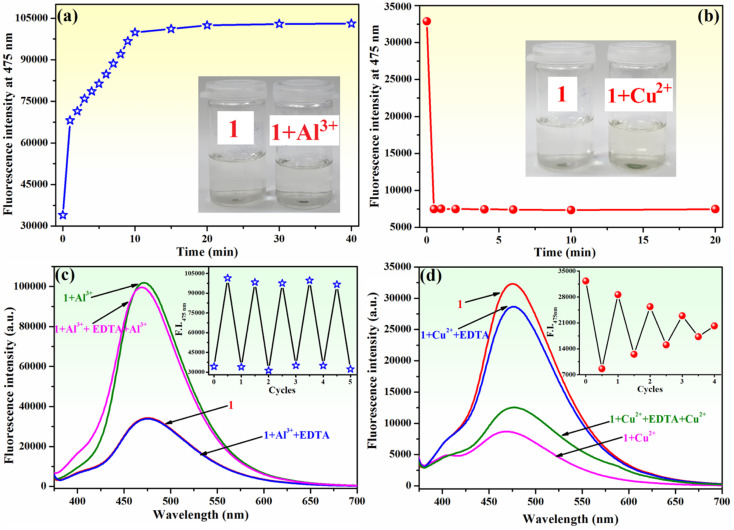
Time-dependent fluorescence intensity (at 475 nm) of sensor 1 (10^−4^ M) in the presence of (a) Al^3+^ (2 equivalents) and (b) Cu^2+^ (2 equivalents) ions in methanol–water (85 : 15, v/v) (*λ*_ex_ = 365 nm). Inset shows images of the solution of 1 before and after the addition of 2 equivalents of either Al^3+^ or Cu^2+^. Emission spectra of 1 (10^−4^ M) after successive cycles of the addition of (c) Al^3+^ and EDTA and (d) Cu^2+^ and EDTA. Inset presents the chemically reversible fluorescence response (at 475 nm) after successive cycles of addition of either Al^3+^ or Cu^2+^ (2 equivalents) and EDTA (2 equivalents) to sensor 1 (10^−4^ M).

The response time of sensor 1 towards Al^3+^ and Cu^2+^ ions was determined. The fluorescence emission of compound 1 (10^−4^ M) increased upon the gradual addition of Al^3+^ ions (2 equivalents within 1 minute), and it continued to intensify for an additional 10 minutes until it reached a stable value that remained unchanged for at least 30 minutes (Fig. 8a). Furthermore, the inset of Fig. 8a demonstrates that the addition of Al^3+^ to the solution of 1 does not result in a discernible colour change. However, the fluorescence quenching of 1 (10^−4^ M) upon the addition of Cu^2+^ ions (2 equivalents) is achieved within 30 seconds, and the attained value is stable and remains unchanged for 20 minutes (Fig. 8b). Upon the addition of Cu^2+^ ions to the solution of compound 1, a slight colour change from colourless to pale yellow was observed (inset of Fig. 8b). The results of this experiment demonstrate that compound 1 exhibits a relatively short sensing response time, requiring 10 minutes to detect Al^3+^ ions and only 30 seconds for Cu^2+^ ions.

The reusability of a probe is a significant feature for the development of chemosensors for practical applications. To ascertain the chemical reversibility of binding of either Al^3+^ or Cu^2+^ to compound 1, EDTA (2 equivalents) was added to 1–Al^3+^ (Fig. 8c) and 1–Cu^2+^ (Fig. 8d) systems. The alternate addition of Al^3+^ and EDTA to the solution of 1 resulted in a reversible “on–off–on” switching fluorescence behaviour for compound 1. The addition of EDTA to the 1–Al^3+^ system induces a reduction of fluorescence intensity at 475 nm almost to the initial value (Fig. 8c). This indicates that the 1–Al^3+^ system undergoes decomplexation due to the formation of a stronger complex between Al^3+^ and EDTA. The fluorescence intensity increases again upon the addition of fresh Al^3+^ ions in the solution of the chemosensor. At least five of these reversible cycles with only a slight loss in fluorescence intensity were carried out (inset of Fig. 8c). This experiment clearly shows that the complex formation in 1 with Al^3+^ is reversible. The reversibility experiment conducted for Cu^2+^ binding to 1 revealed that the addition of EDTA (2 equivalents) to 1–Cu^2+^ system allows for a recovery of the fluorescence intensity of compound 1 at 475 nm to approximately 88% of its original value (Fig. 8d). Further addition of Cu^2+^ ions results in quenching of fluorescence. However, this process is less pronounced than that observed during the initial use cycle. The alteration in fluorescence intensity recovery and sensitivity for Cu^2+^ detection is observed during four cycles of sequential addition of Cu^2+^ ions and EDTA (inset of Fig. 8d). Therefore, the complexation behaviour of 1 to Cu^2+^ is not fully reversible, resulting in a notable decline in the ability of compound 1 to detect Cu^2+^ during several cycles of utilization.

As compound 1 can act as a selective, sensitive and reversible fluorescence sensor for Al^3+^, its reliability in the detection of Al^3+^ in different samples using the linear regression equation given above (*I* = 3834.88 × *C*_Al_^3+^ + 37 447.17) was examined. The values determined for Al^3+^ concentration from the investigated samples are presented in Table 1, together with the fluorescence recovery of compound 1. These studies confirm the ability of compound 1 to serve as an effective chemosensor for the detection of Al^3+^ ions in real samples without interference from other ions that may be present in these samples.

**Table 1:** Detection of Al^3+^ by compound 1 (10^−4^ M) in real samples

Sample	Al^3+^ spiked (M)	Al^3+^ found (M)	Recovery (%)
Millipore water	0	0	—
	5.00 × 10^−5^	4.87 × 10^−5^	100.5
Tap water	0	2.87 × 10^−6^	98.9
	5.00 × 10^−5^	5.19 × 10^−5^	99.2
Soft drink from a plastic bottle	0	0	—
	5.00 × 10^−5^	4.95 × 10^−5^	101.1
Soft drink from an aluminum can	0	3.91 × 10^−6^	97.4
	5.00 × 10^−5^	5.02 × 10^−5^	98.3

### 3.5. Chemosensing ability of phenolic Mannich base 1 for fluorescent detection of rare earth metal ions

Rare earth metal (lanthanide) ions are important components of many technological devices and are also used in medical diagnostic applications. For these reasons, scientists around the world have become more interested in research involving rare earth elements, particularly in the search for chemical sensors capable of sensing trivalent lanthanide ions.^80^ The coordination involving rare earth cations is strongly influenced by their 4f electronic configuration, which leads to a complicated behavior in coordination processes, and makes them in turn significantly different from the other metal ions examined in this study. In this context, we chose to investigate the possibility for phenolic Mannich base 1 to function as a fluorescence sensor for rare earth metal ions.

**Fig. 9:**
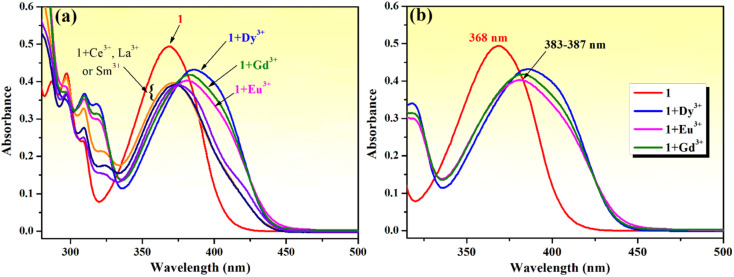
(a) Change in the absorption spectrum of compound 1 (10^−4^ M) in the presence of different rare earth metal ions (5 equivalents of La^3+^, Ce^3+^, Eu^3+^, Dy^3+^, Sm^3+^ and Gd^3+^) in methanol–water (85 : 15, v/v). (b) Details of absorption maximum shifts for phenolic Mannich base 1 (10^−4^ M) upon addition of Eu^3+^, Dy^3+^ and Gd^3+^ ions (5 equivalents).

First, the chemosensing ability of compound 1 toward a series of rare earth metal ions comprising La^3+^, Ce^3+^, Eu^3+^, Dy^3+^, Sm^3+^ and Gd^3+^ was evaluated through UV-vis spectroscopy. As shown in Fig. 9a, the absorption spectrum of compound 1 is modified upon the addition of lanthanide ions (5 equivalents). The presence of La^3+^, Ce^3+^ and Sm^3+^ ions leads to a decrease in the absorption intensity and a broadening of the absorption band, and the presence of Eu^3+^, Dy^3+^ and Gd^3+^ causes the absorption maximum to shift and slightly reshapes the absorption curve profile. The addition of Eu^3+^, Dy^3+^ and Gd^3+^ ions makes the absorption peak redshift from 368 nm to the range of 383–387 nm, while it also decreases the absorption intensity and broadens the absorption band (Fig. 9b). These changes suggest the formation of charge transfer complex compounds between these lanthanide ions and Mannich base 1.

**Fig. 10:**
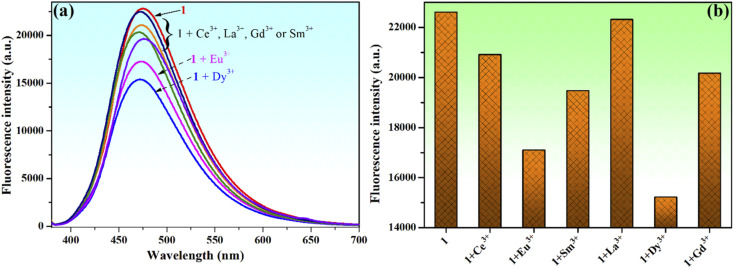
Change in the fluorescence spectrum (a) and fluorescence intensity (b) of compound 1 (10^−4^ M) upon mixing with different rare earth metal ions (2 equivalents of La^3+^, Ce^3+^, Eu^3+^, Dy^3+^, Sm^3+^ and Gd^3+^) in methanol–water (85 : 15, v/v) when *λ*_ex_ = 365 nm.

**Fig. 11:**
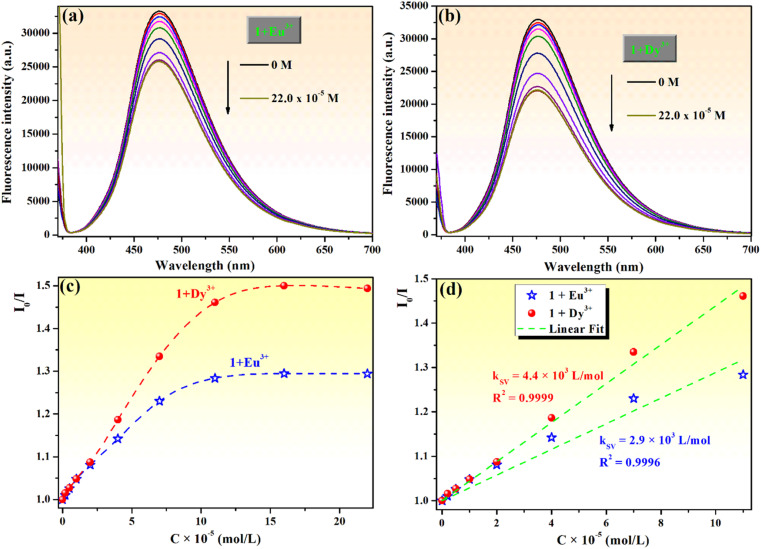
Fluorescence spectra of compound 1 (10^−4^ M) upon addition of different concentrations (0–2.2 equivalents) of (a) Eu^3+^ and (b) Dy^3+^ ions. Stern–Volmer plots (c) and linear fit of Stern–Volmer plots in the concentration range of 0–11 × 10^−5^ M (d) for the interactions between compound 1 and Eu^3+^ or Dy^3+^ ions (at *λ*_ex_ = 365 nm).

Moreover, the chemosensing ability of compound 1 (10^−4^ M) for selected rare earth metal ions (La^3+^, Ce^3+^, Eu^3+^, Dy^3+^, Sm^3+^ and Gd^3+^) was investigated by fluorescence measurements, revealing that only the addition of Eu^3+^ or Dy^3+^ ions has important quenching effects on the fluorescence emission of compound 1 (Fig. 10a and b). Upon addition of 2 equivalents Eu^3+^ to the solution of sensor 1, the fluorescence intensity of phenolic Mannich base 1 is quenched by 24.4%, while the quenching produced by the addition of the same concertation of Dy^3+^ ions is 32.7%. Even if the fluorescence response of sensor 1 toward Eu^3+^ and Dy^3+^ ions is rather weak, the fluorescence quenching behavior of 1 upon the gradual addition of increasing amounts of aqueous solutions of Eu^3+^ or Dy^3+^ (0–2.5 equivalents) was further investigated (Fig. 11).

Compound 1 exhibits fluorescence quenching in the presence of Eu^3+^ and Dy^3+^ ions up to a concentration of 16.0 × 10^−5^ M (1.6 equivalents). Beyond this threshold, there is no discernible effect of the increasing concentration of the rare earth ions on the intensity of fluorescence of sensor 1 (Fig. 11a and b). These findings are even better illustrated by the Stern–Volmer representations (Fig. 11c), which also show that compound 1 presents an increased sensitivity toward the detection of Dy^3+^ through fluorescence quenching. The detection limits of compound 1 for Eu^3+^ and Dy^3+^, calculated with eqn (1), are 1.42 × 10^−5^ M and 0.99 × 10^−5^ M, respectively. Hence, phenolic Mannich base 1 can be used as a fluorescent chemosensor with a “turn-off” response for Eu^3+^ and Dy^3+^ ions only in a narrow concentration range, specifically 0.99–16.0 × 10^−5^ M for Dy^3+^ and 1.42–16.0 × 10^−5^ M for Eu^3+^. The Stern–Volmer graphs for systems 1–Eu^3+^ and 1–Dy^3+^ present a linear domain, followed by downward curvature (Fig. 11c), indicating that a parallel quenching mechanism occurs (*e.g.*, energy transfer and ground state complex formation) in addition to dynamic quenching. For both lanthanide ions, the Stern–Volmer constants (*K*_SV_) were determined (Fig. 11d) considering the linear range (0–11.0 × 10^−5^ M) of the graphs as 4.4 × 10^3^ L mol^−1^ for Dy^3+^ (*R*^2^ = 0.9999) and 2.9 × 10^3^ L mol^−1^ for Eu^3+^ (*R*^2^ = 0.9996). Because the Stern–Volmer constant for Dy^3+^ ions is slightly greater than that of the Eu^3+^ quencher, it can be inferred that compound 1 has a better sensitivity for Dy^3+^ ions over the other lanthanide ions evaluated in this study and has the potential to be used for the fluorescence-based detection of Dy^3+^ ions.

## 4 Conclusions

This study reports the synthesis of an aminomethylated derivative produced through the use of 1-(1-hydroxynaphthalen-2-yl)ethanone in the Mannich reaction as a substrate that features a particular *ortho*-hydroxyphenone scaffold along with its unprecedented application as a chemosensor. The aminomethylation of HAN produced a novel compound whose quantum yield was slightly improved compared to that of the starting material. The evaluation of the ability of a large number of cations to influence the fluorescence response of this phenolic Mannich base allowed the discrimination of Al^3+^ as a potent fluorescence enhancer, and the identification of Cu^2+^ as an effective fluorescence quencher. Although this chemosensor was sensitive to these cations, competition experiments proved that the phenolic Mannich base under investigation was selective toward Al^3+^. Out of several rare earth metal ions, only Eu^3+^ or Dy^3+^ elicited a weak response from the sensing molecule, which acts as a fluorescence quencher. The interesting results presented in this study require further investigations within this line of research that should, on the one hand, examine the potential as chemosensors for cations of derivatives of this phenolic Mannich bases obtained through the chemical modification of the carbonyl function (*i.e.*, its oxime, hydrazone, thiosemicarbazone, *etc.*), or of analogs of this phenolic Mannich base derived from amines other than morpholine (preferably with a structure that generates a secondary recognition site for cations and enables cation binding and complex formation), or, on the other hand, expand the current study toward the detection of other analytes besides cations, such as small organic molecules (nitroaromatics, endogenous and xenobiotic thiols, nucleotides, *etc.*) or inorganic compounds (hydrazine and hydrogen peroxide).

## Data availability

Large sets of experimental data are available from the corresponding author. The data that support the findings of this study will be made available on request.

## Author contributions

Andreea Laura Chibac-Scutaru: conceptualization, investigation, data curation, visualization, writing – original draft, writing—review & editing. Gheorghe Roman: conceptualization, investigation, writing – original draft, writing—review & editing. All authors have read and approved the published version of the manuscript.

## Conflicts of interest

The authors declare that they have no known competing financial interests or personal relationships that could have appeared to influence the work reported in this paper.

## Supplementary Material

RA-014-D4RA07200F-s001
