# Supplementary material for: Fluorescence sensing of metal ions in solution using a morpholine-containing phenolic Mannich base of 1′-hydroxy-2′-acetonaphthone
Source: RSC Adv. 2024 Dec 6;14(52):38590–604. doi: 10.1039/d4ra07200f (PMC11622784; doi:10.1039/d4ra07200f)
Supplement: RA-014-D4RA07200F-s001 [file RA-014-D4RA07200F-s001.pdf]

Supplementary Information (SI) for RSC Advances.
This journal is © The Royal Society of Chemistry 2024

Supplementary Information

Fluorescence sensing of metal ions in solution by a morpholine-containing phenolic Mannich base of 1′-hydroxy-2′-acetonaphthone

Andreea Laura Chibac-Scutaru[1,*], Gheorghe Roman[2]

[1]Petru Poni Institute of Macromolecular Chemistry, Department of Polyaddition and Photochemistry, Iaşi, 700487, Romania; andreea.chibac@icmpp.ro; ORCID: 0000-0003-3503-0237.

[2]Petru Poni Institute of Macromolecular Chemistry, Department of Inorganic Polymers, Iaşi, 700487, Romania; gheorghe.roman@icmpp.ro; ORCID: 0000-0003-0939-1899.

*Correspondence: andreea.chibac@icmpp.ro (ALCS).

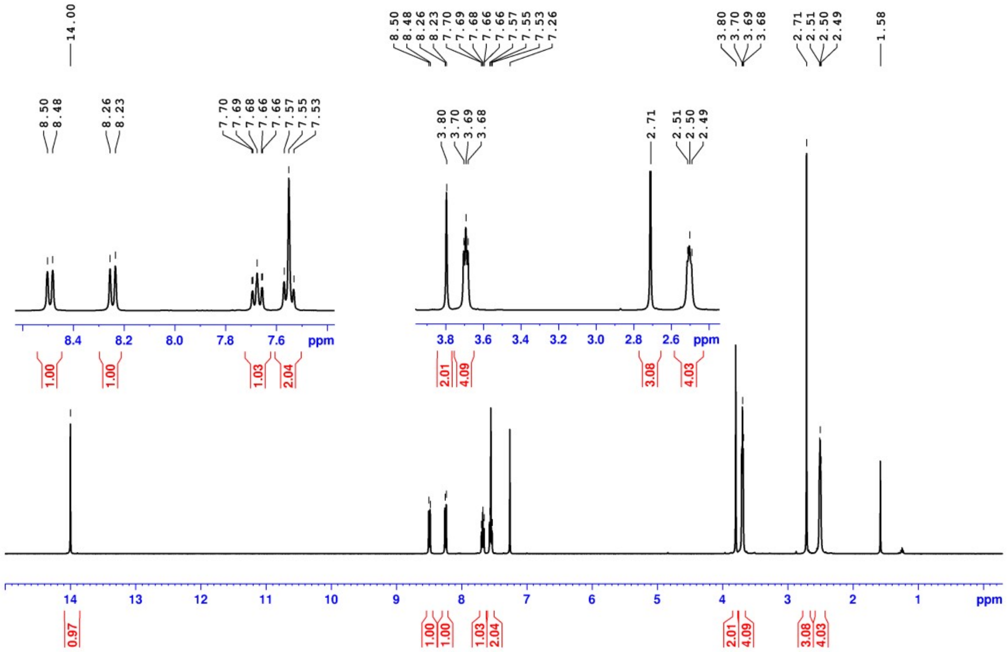

Figure S1. ^{1}H NMR spectrum of compound **1**.

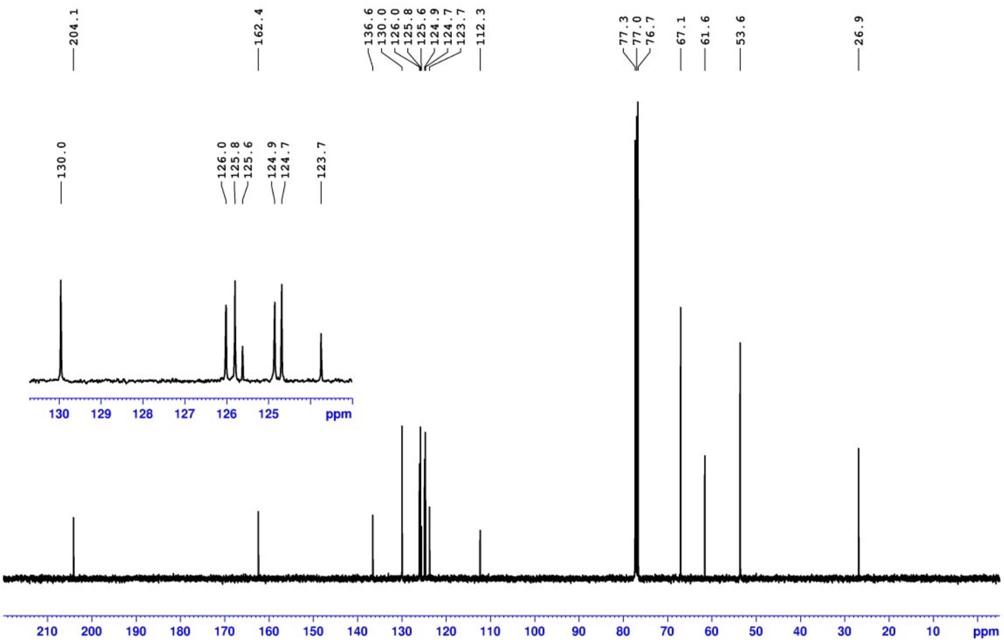

Figure S2. ^{13}C NMR spectrum of compound **1**.

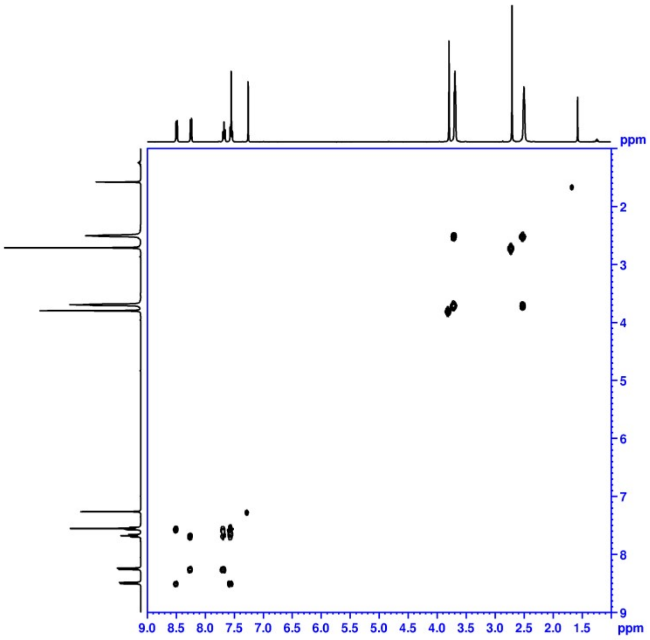

Figure S3. H,H-COSY spectrum of compound **1**.

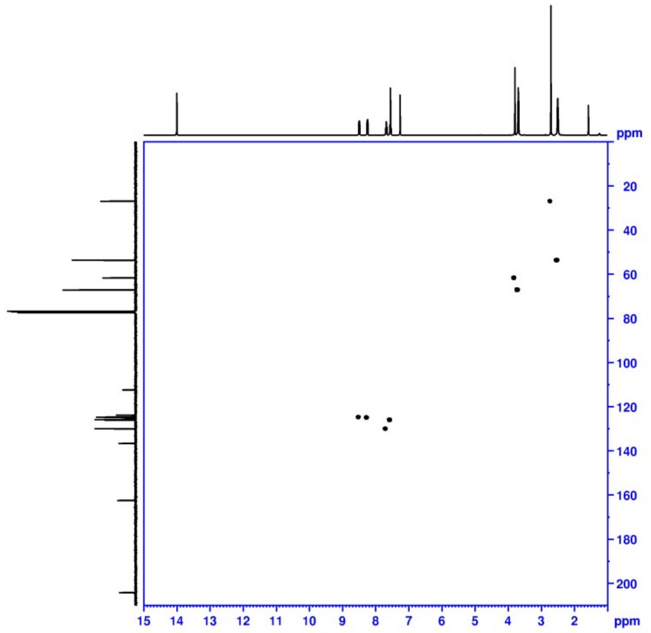

Figure S4. H,C-HSQC spectrum of compound **1**.

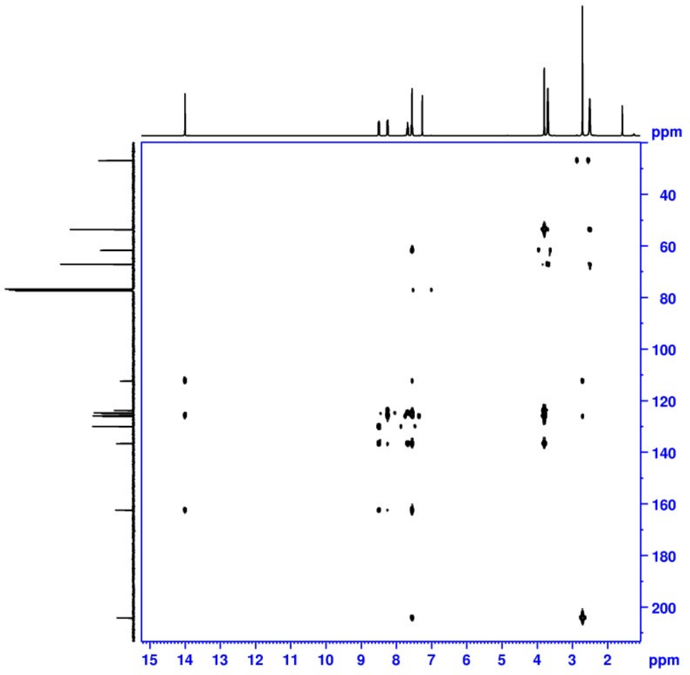

Figure S5. H,C-HMBC spectrum of compound **1**.

Table S1. Photophysical properties of compound **1** compared to those of HAN in methanol.

compound	λ_{abs} (nm)	ε (M^{-1} cm^{-1})[a]	λ_{em} (nm)	Φ (%)
**1**	287; 298; 310; 368	5158	475	2.91
**HAN**	283; 293; 305; 363	4385	473	2.44

[a]The value for the molar extinction coefficient (ε) is determined for compound **1** at λ_{abs} = 368 nm and for HAN at λ_{abs} = 363 nm.

Table S2. Decay components and their contribution to the fluorescence lifetime of compound **1** (10^{-4} M) in presence of different Al^{3+} concentrations. The decays were monitored at 475 nm (λ_{exc}= 375 nm).

[Al^{3+}] (M)	τ_1 (ns)	A_1 (%)	τ_2 (ns)	A_2 (%)	<τ_F> (ns)*
0	0.2383	83.12	4.1509	16.88	3.2886
0.5×10^{-4}	0.2943	39.51	6.9943	60.49	6.8151
1.0×10^{-4}	0.3791	30.41	8.2081	69.59	8.0532
1.5×10^{-4}	0.4469	22.70	8.4847	77.30	8.3623
2.0×10^{-4}	0.5751	20.66	8.6751	79.34	8.6275
2.5×10^{-4}	0.7973	18.96	8.8093	81.04	8.6432

*The effective average lifetime (τ_F) was calculated using the equation given in J.R. Lakowicz, Principles of Fluorescence Spectroscopy, Springer, Boston, 2006:

$$< \tau_F > = \frac{(A_1(\tau_1)^2 + A_2(\tau_2)^2}{A_1\tau_1 + A_2\tau_2}$$

Table S3. Decay components and their contribution to the fluorescence lifetime of compound **1** (10^{-4} M) in presence of different Cu^{2+} concentrations. The decays were monitored at 475 nm (λ_{exc}= 375 nm).

$[Cu^{2+}]$ (M)	τ_1 (ns)	A_1 (%)	τ_2 (ns)	A_2 (%)	$<\tau_F>$ (ns)*
0	0.2383	83.12	4.1509	16.88	3.2886
0.5×10^{-4}	0.2419	74.63	4.70054	25.37	4.1190
1.0×10^{-4}	0.2912	66.45	5.3647	33.55	4.8722
1.5×10^{-4}	0.2948	64.57	5.3321	35.43	4.8710
2.0×10^{-4}	0.2904	64.00	5.4405	36.00	4.9941
2.5×10^{-4}	0.2948	64.11	5.5568	35.89	5.1013

*The effective average lifetime (τ_F) was calculated using the equation given in J.R. Lakowicz, Principles of Fluorescence Spectroscopy, Springer, Boston, 2006:

$$< \tau_F > \ = \frac{(A_1(\tau_1)^2 + A_2(\tau_2)^2}{A_1\tau_1 + A_2\tau_2}$$

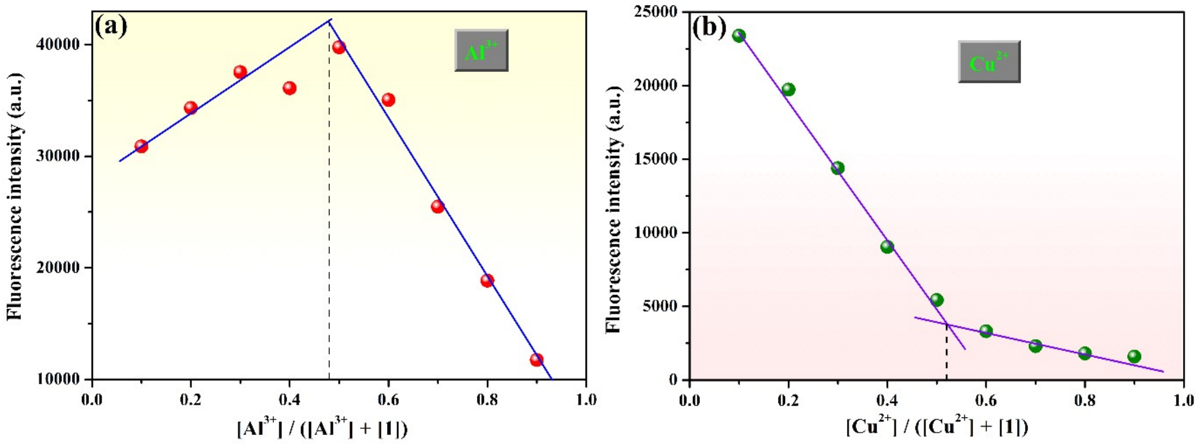

Figure S6. Job's plot for complexation of compound **1** with (a) Al^{3+} and (b) Cu^{2+} in methanol–water (85:15, *v/v*).

Determination of association constant (K_a)

The association constant (K_a) was obtained from fluorescence titration data according to the Benesi–Hildebrand equation:

$$\frac{1}{I - I_0} = \frac{1}{K_a[C](I_{max} - I)} + \frac{1}{I_{max} - I_0}$$

where, I is fluorescence intensity of compound **1** (λ_{em} = 475 nm) in the presence of a given concentration of Al^{3+}, I_0 and I_{max} represents initial fluorescence intensity of sensor **1** in the absence of Al^{3+} and the fluorescence intensity of sensor **1** in the presence of the highest concentration of Al^{3+}, respectively; K_a is the association constant, and C is the concentration of Al^{3+}.

For fluorescence quenching in presence of Cu^{2+}, a slightly modified equation was used:

$$\frac{1}{I - I_0} = \frac{1}{K_a[C](I_{min} - I_0)} + \frac{1}{I_{min} - I_0}$$

where I_{min} is intensity of compound **1** in the presence of the highest concentration of Cu^{2+}.

The values of intercept and slope were taken from the plot of $(I_{max} - I_0) / (I - I_0)$ *versus* $1/[Al^{3+}]$ for **1**–Al^{3+} complex (Fig. S7a) and from the plot of $(I_{min} - I_0) / (I - I_0)$ *versus* $1/[Cu^{2+}]$ for **1**–Cu^{2+} complex (Fig. S7b).

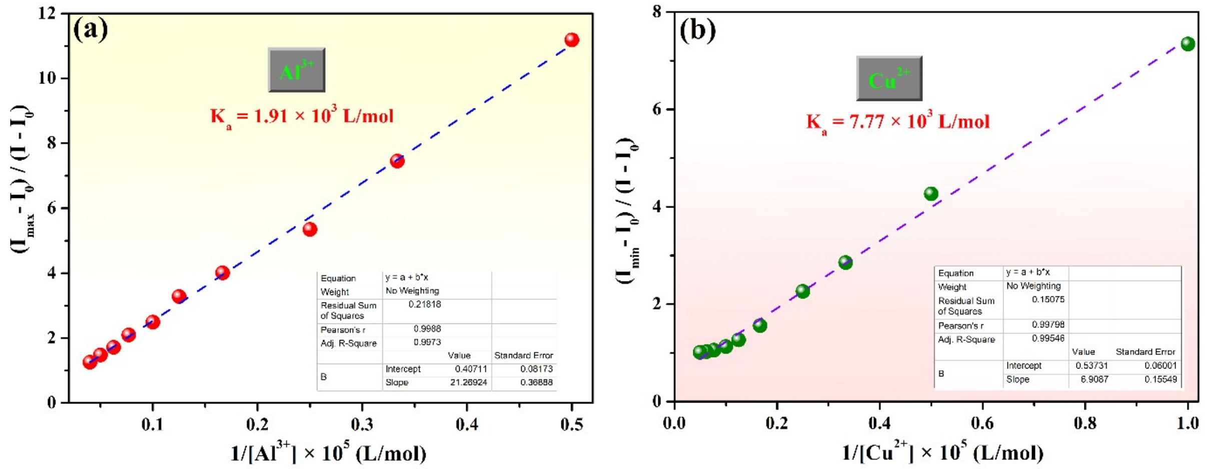

Figure S7. Benesi–Hildebrand plots for the determination of the association constants (K_a) for the complexation of compound **1** (10^{-4} M) with (a) Al^{3+} and (b) Cu^{2+}.
